# Give Rabies and Hydrophobia a Rest

**Published:** 1900-06

**Authors:** 


### GIVE RABIES AND HYDROPHOBIA A REST.

Of all the subjects exhaustively written upon by scientific writers for periodicals and books, bulletins and reports, by space writers and reporters for the yellow journals, and through every other avenue of the pen, to strike terror and fear to the average mind of laymen, and to even make shudder those engaged in the pursuit of scientific medicine, as they read in endless pages of manuscript and print the history and description of outbreaks so varying in character and aspect as have followed in the wake of " rabies and hydrophobia " from time immemorial. The never ceasing crusade against dogs, the wonderful results obtained through the use of madstones, the use of charms, and occult remedies, faith cures, fake Pasteur institutes, would fill books of thousands of pages, so that one would be led to believe that these diseases were as common almost as tuberculosis; while in truth the average veterinarian lives and dies without ever observing a single case in the human family, and only knows of them through sensational newspaper reports spread all over the land to add terror and fear to the timid mind. Is it not about time to give this subject a rest until more real scientific work is done in this field of research and some more accurate work completed beyond those tests on rabbits and guinea-pigs, so lacking in conclusiveness to a majority of scientific minds? A disease so rare if known at all among those most exposed to its virility—the dog catchers of every city and town—is one that should impress everyone learned in the profession of medicine to consider very deeply from a prophylactic point of view, and be ever ready to preserve and protect the human family from pseudophobia or lyssophobia that follow in the wake of mad-dog scares, widespread quarantine protection, multiplication of Pasteur institutes, discoveries of more effective madstones, and every other conceivable plan of treatment to play upon the minds of weak and incredulous people.

The veterinarian has an important duty to perform in this work, and he should lead in the control of these uncalled for and unfounded injurious reports that cloud scientific pathology.

The Italian veterinary profession suffered a serious loss in February, 1900, by the death of Professor Giovanni Canestini, Professor of Zoölogy and Comparative Anatomy. He was very active in the Society of Natural Sciences of Padova, Italy.

---

On May 1st the Missouri and Kansas State Sanitary Boards inaugurated the tuberculin test requirements in their respective States. A certificate from a qualified veterinarian or an examination at points of receipt and shipment are now required for all breeding and dairy stock destined to remain within the State limits. The result of such concerted movements will soon be strongly apparent.

